# Case report: persistent fifth aortic arch presenting as a giant ascending aortic aneurysm: diagnostic challenge and surgical resolution with Bentall procedure

**DOI:** 10.1093/ehjcr/ytag330

**Published:** 2026-05-12

**Authors:** Abdelrahman Waleed Alsayed Manaa, Mokhtar Kahin, Mukhtar Metwally, Rafat Abu Shakra, Walid Abu Khudair

**Affiliations:** Cardiac Surgery Department, International Medical Center (IMC), PO Box 2172, Jeddah 21451, Saudi Arabia; Cardiac Surgery Department, International Medical Center (IMC), PO Box 2172, Jeddah 21451, Saudi Arabia; Cardiac Surgery Department, International Medical Center (IMC), PO Box 2172, Jeddah 21451, Saudi Arabia; Pathology and Laboratory Medicine Department, International Medical Center (IMC), PO Box 2172, Jeddah 21451, Saudi Arabia; Cardiac Surgery Department, International Medical Center (IMC), PO Box 2172, Jeddah 21451, Saudi Arabia

**Keywords:** Persistent fifth aortic arch, Aortic aneurysm, Aortic dissection, Bentall procedure, Giant aneurysm, Cardiothoracic surgery, Case report

## Abstract

**Background:**

An ascending aortic aneurysm is defined as dilatation of the ascending aorta to ≥1.5 times its normal diameter. Surgical intervention is recommended when the diameter exceeds 55 mm to prevent life-threatening complications such as rupture or dissection. Persistent fifth aortic arch is a rare congenital vascular anomaly, which may present as a dual-lumen aortic configuration and can mimic acquired pathological conditions such as aortic dissection or aneurysm, posing a diagnostic challenge.

**Case summary:**

A 39-year-old male with long-standing hypertension presented with cough and shortness of breath. Initial computed tomography (CT) performed to exclude pulmonary embolism incidentally revealed a markedly dilated ascending aorta. Following intensive care unit admission and stabilization, echocardiography and CT aortography demonstrated severe aortic regurgitation and a giant ascending aortic aneurysm measuring 10.5 cm with dual-lumen morphology interpreted radiologically as Stanford type A aortic dissection. The patient underwent urgent Bentall procedure. Histopathological examination confirmed a dual-lumen vascular structure consistent with persistent fifth aortic arch, establishing the congenital aetiology. The patient recovered uneventfully and remained asymptomatic during cardiothoracic follow-up.

**Conclusion:**

This case highlights the diagnostic challenge of distinguishing congenital persistent fifth aortic arch from acquired aortic dissection in the setting of a giant ascending aneurysm. Despite radiologic ambiguity, prompt surgical repair was definitive. Histopathological confirmation provided etiologic clarity and contributes to understanding the natural history of this rare anomaly.

Learning points

- Dual-lumen ascending aortic anatomy from a persistent fifth aortic arch may appear radiologically identical to chronic aortic dissection, complicating accurate diagnosis.
- Early recognition of congenital aortic variants and prompt surgical management are vital in preventing life-threatening complications in giant ascending aortic aneurysms.

## Introduction

An ascending aortic aneurysm (AAA) is defined as a localized or diffuse dilatation of the ascending segment of the aorta, including the aortic root or tubular segment, that exceeds 1.5 times the normal diameter.^1^ According to the 2022 ACC/AHA Aortic Disease Guidelines, ascending aortic aneurysms carry significant risk of rupture, dissection, and valve dysfunction, and early surgical intervention is recommended when size thresholds are exceeded.^1^ The Bentall procedure, involving replacement of the aortic root and ascending aorta with a composite graft remains the gold standard technique.^2,3^

We present a case of a giant (10.5 cm) AAA in a young adult man, with a dual-lumen configuration that was initially interpreted radiologically as a Stanford type A dissection, which demonstrated features consistent with a persistent fifth aortic arch (PFAA) variant, suggesting a rare congenital vascular anomaly. This report emphasizes the diagnostic challenges, surgical considerations, and post-operative follow-up in managing life-threatening conditions, highlighting the importance of timely surgical intervention.

## Summary figure

**Figure:**
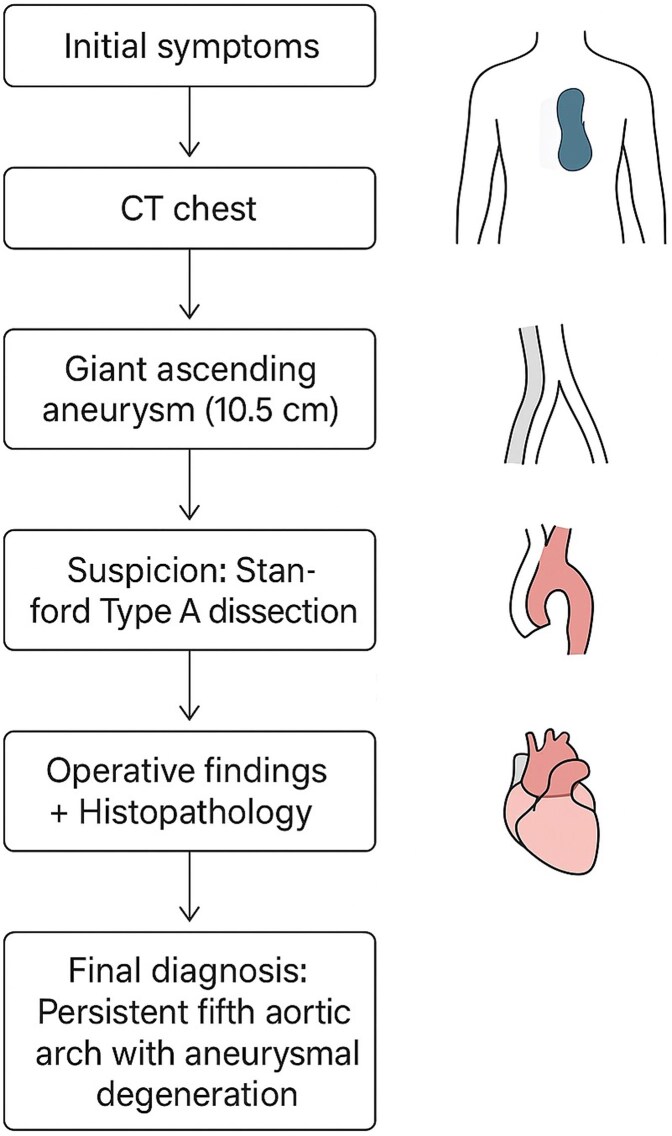


## Case presentation

### Initial presentation and diagnostic workup

A 39-year-old Middle Eastern male with a history of hypertension treated with amlodipine/valsartan/hydrochlorothiazide (160/10/25 mg), obesity, heavy smoking, and lumbar disc prolapse presented to the emergency department with progressive shortness of breath associated with cough. He reported recent exposure to respiratory illness among family members. On examination, the patient was conscious and alert, with hypoxia (oxygen saturation 85%), tachycardia, and elevated blood pressure. Cardiac examination was not documented while chest examination revealed decreased air entry at the basal zones bilaterally, with fine basal crepitations. The remainder of the physical examination was unremarkable. No Marfanoid features or clinical stigmata of connective tissue disorders were identified. Initial management with oxygen supplement improved O_2_ saturation to 95%.

Given the acute respiratory presentation and hypoxia, pulmonary embolism was suspected, and computed tomography pulmonary angiography was performed. Pulmonary embolism was excluded; however, imaging demonstrated bilateral patchy ground-glass opacities consistent with an infectious or inflammatory process. Importantly, the computed tomography (CT) scan also revealed a markedly dilated ascending aorta consistent with a large aneurysm. In the context of hypoxia and this incidental but critical vascular finding, the patient was referred to the cardiology service for further evaluation and management.

Electrocardiography demonstrated sinus tachycardia with non-specific changes. Laboratory investigations showed no evidence of myocardial injury (troponin 0.01 ng/ml; normal <0.04 ng/ml), preserved renal function, and mildly elevated inflammatory markers (C-reactive protein 15 mg/L; normal <5 mg/L). The patient was admitted to the cardiac care unit for close monitoring and further diagnostic workup.

Urgent transthoracic echocardiography on the first day upon admission demonstrated preserved ventricular function with severe aortic regurgitation associated with marked aortic root dilation.

The patient developed worsening hypoxia consistent with type I respiratory failure, requiring escalation of oxygen therapy to high-flow nasal cannula. Infectious causes were suspected, and broad-spectrum antibiotics and corticosteroid therapy were initiated. Strict blood pressure control was also implemented to reduce aortic wall stress.

**Figure 1:**
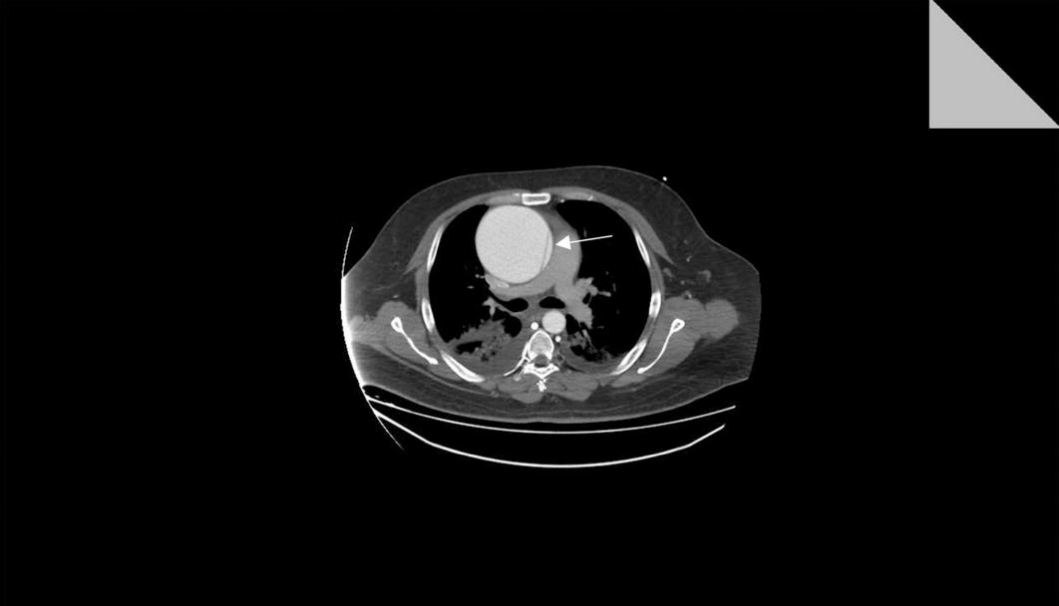
A large aneurysm is seen involving the whole ascending aorta with a maximum diameter of 10.5 cm. A dissecting flap is seen starting at the upper part of the ascending aorta and extending to the aortic arch.

Following respiratory stabilization on the second day, dedicated CT aortography was performed, confirmed a giant ascending aortic aneurysm measuring approximately 10.5 cm in maximal diameter, with features interpreted radiologically as type A aortic dissection (*Figure 1*). Cardiothoracic surgical consultation was obtained, and urgent surgical intervention was recommended due to the high risk of rupture and progressive haemodynamic compromise.

Despite detailed counselling regarding the life-threatening risk of rupture and complications, the patient initially declined surgical intervention. Strict blood pressure and heart rate control was maintained. However, given the aneurysm size, associated severe aortic regurgitation, and overall risk profile, surgical repair was ultimately pursued on the third day after informed consent was obtained.

### Diagnostic modality

#### Echocardiographic assessment

Transthoracic echocardiography demonstrated normal bi-ventricular size and functions with no regional wall motion abnormalities. Mild concentric left ventricular hypertrophy and mild left atrial dilation were noted. The aortic valve was trileaflet in morphology. The aortic root was severely aneurysmal, measuring 9.8 cm, with associated severe aortic regurgitation and the left ventricular outflow tract diameter measured 29 mm. No additional valvular abnormalities were identified. Estimated pulmonary artery systolic pressure was 30 mmHg.

#### CT aortic angiography

CT aortography demonstrated large ascending aortic aneurysm of 10.5 cm in maximal diameter with dissection flap involving upper part of ascending aorta to proximal brachiocephalic artery. No mural thrombus, wall calcification, or signs of impending rupture were observed. The major aortic branches were patent and of normal calibre. These findings were interpreted radiologically as Stanford type A aortic dissection.

#### Surgical approach

The patient underwent urgent surgical repair under general anaesthesia. Following median sternotomy and institution of cardiopulmonary bypass, the ascending aorta was opened above the sinotubular junction. Intraoperative inspection revealed marked aneurysmal dilation of the aortic root and ascending aorta with involvement of the sinuses of Valsalva. A prominent intraluminal flap was noted. No mural thrombus was identified. The native aortic valve leaflets exhibited multiple perforations and unsuitable for preservation; therefore, a Bentall procedure was performed using a 29-mm composite valved conduit. The diseased aortic root and ascending aorta were excised and the coronary arteries were reimplanted as coronary buttons. The distal graft was anastomosed to the proximal aortic arch under brief hypothermic circulatory arrest. Excised tissue was sent for histopathology examination. The patient was successfully weaned from cardiopulmonary bypass, and intraoperative transoesophageal echocardiography confirmed a well-functioning prosthetic valve with no residual aortic regurgitation and preserved ventricular function.

#### Histopathological findings

**Figure 2:**
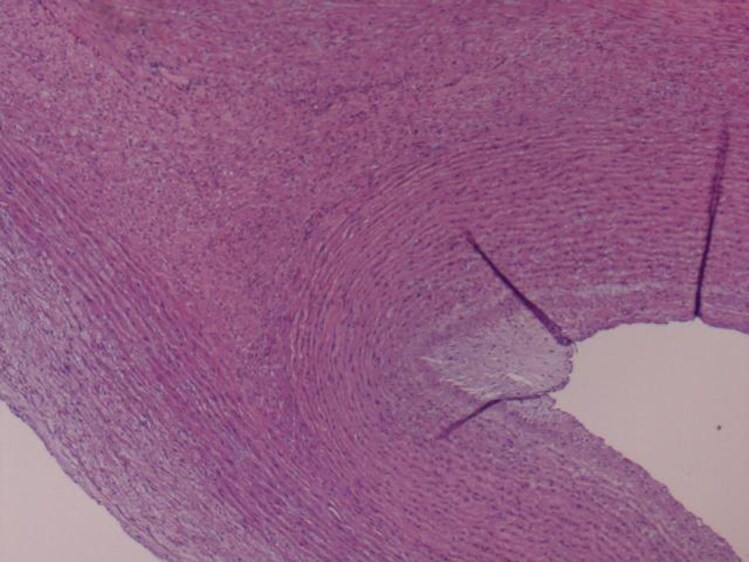
Microscopic examination revealed two vascular lumens; the larger is present at the left lower angle, lined by endothelial cells, tunica intima, and tunica media; the smaller lumen is present at the right aspect of the photo, lined by endothelial cells, tunica intima, and tunica media. The smooth muscle fibres are arranged concentrically around the small vascular lumen in keeping with the fifth primitive aortic arch remnant (H&E stain, 40 × magnification).

**Figure 3:**
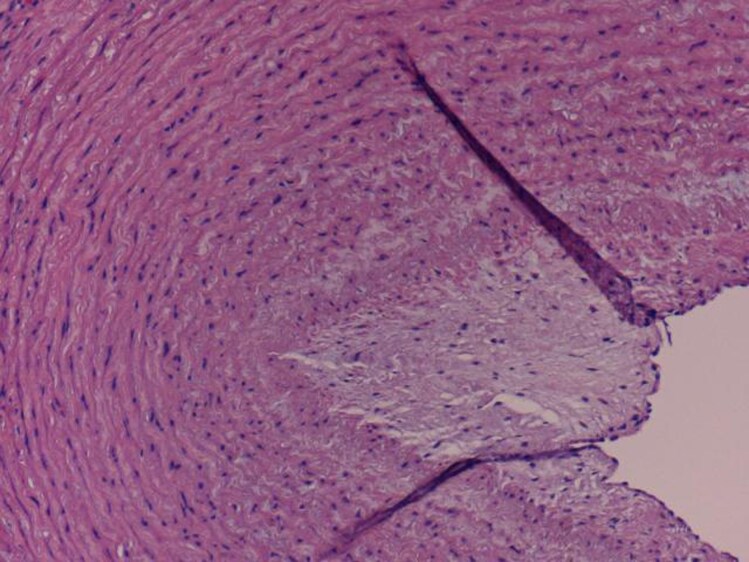
Higher magnification showing the smaller vascular lumen of the fifth primitive aortic arch remnant lined by endothelial cells, tunica intima, and tunica media with concentric arrangement of smooth muscle fibres around the vascular lumen (H&E stain, 100 × magnification).

**Figure 4:**
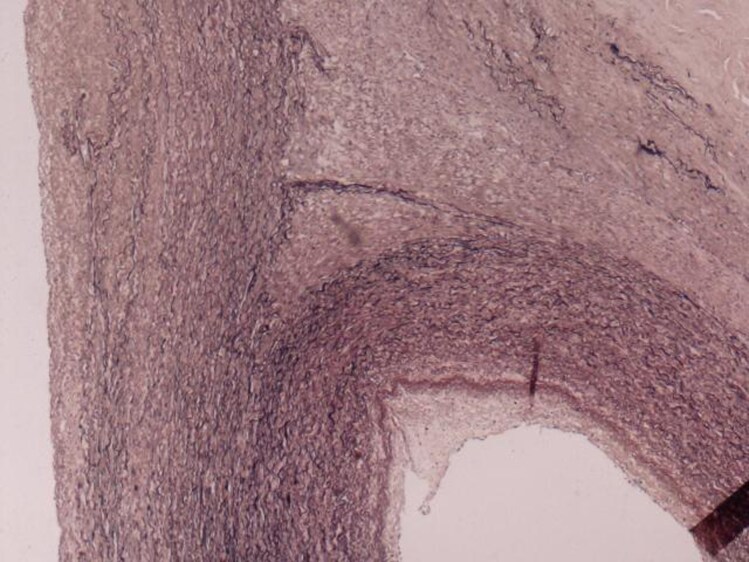
Elastic Verhoeff Van Gieson stain highlights the concentric arrangement of elastic fibres around the small vascular lumen of the fifth primitive aortic arch remnant at the right lower aspect of the photo (40 × magnification).

The excised aortic segment measured 7.5 cm in length and 9.0 cm in diameter and contained a smaller lumen measuring 2.5 × 3.5 cm. Histopathological examination revealed two lumens, one corresponding to the true lumen and the other smaller lumen displaying a well-formed vascular wall with an endothelial lining, intima, and muscular media, along with elastic tissue degeneration and mucinous accumulation. The adventitia showed mild chronic inflammation and fibrosis; these findings favour a congenital anomaly—specifically a PFAA—over an aortic dissection (*Figures 2–4*).

#### Post-operative, discharge, and follow-up

The postoperative course was uneventful, and the patient recovered without complications. Echocardiography confirmed a well-seated, normally functioning mechanical bileaflet aortic valve prosthesis with no paravalvular leak or significant transvalvular gradient. The mean aortic valve gradient ranged from 8 to 11 mmHg. The left ventricle was normal in size and function with mild concentric hypertrophy. The left atrium was mildly dilated, and mild pulmonary hypertension was observed.

The patient was discharged 10 days after the surgery under stable conditions with anticoagulants and antihypertensives. At follow-up visits, the patient demonstrated clinical improvement. Echocardiographic reassessment confirmed preserved left ventricular systolic function, no aortic regurgitation, and stable prosthetic valve function.

#### Patient perspective

The patient, a 39-year-old banker, reported significant concern upon learning of the unexpected diagnosis of a life-threatening aortic condition, particularly given his relatively young age and initial presentation with respiratory symptoms. After detailed discussions with the cardiothoracic team, he agreed to proceed with surgical treatment. During follow-up, he expressed reassurance and satisfaction with the outcome. He has since returned to his professional and daily activities and remains committed to long-term health measures, including strict blood pressure control, routine clinical follow-up, weight management, and regular exercise as part of his recovery and prevention plan.

## Discussion

This report describes a rare and diagnostically challenging presentation of a giant ascending aortic aneurysm (10.5 cm) with dual-lumen morphology, radiologically interpreted as Stanford type A dissection. Histological examination revealed a congenital vascular anomaly, which was likely a persistent PFAA. This case adds several important lessons to the published series on giant aneurysms and surgical root replacement. Importantly, CT aortography demonstrated an intraluminal flap extending from the ascending aorta to the proximal arch and was interpreted radiologically as Stanford type A and DeBakey type I dissection; however, intraoperative findings and histopathological examination confirmed the presence of a dual-lumen vascular structure composed of organized intimal, medial, and endothelial layers, supporting a congenital origin rather than an acquired dissection. This discrepancy highlights the diagnostic limitations of imaging alone and underscores the importance of histopathological confirmation in cases with atypical aortic morphology.

In human embryos, the fifth aortic arch is transient or absent; if it persists, it may form an abnormal connection between the ascending and descending aorta, resulting in dual-lumen or double-barrelled configurations.^4,5^ Although PFAA frequently remains asymptomatic, some cases have been associated with aneurysmal dilation, coarctation, or pseudo-dissection.^6,7^ While the initial imaging findings in this case favoured chronic type A dissection, histopathological evaluation showed two distinct lumina, both endothelialized with intact intimal, medial, and muscular layers, which is inconsistent with dissection, where the false lumen lacked a typical vascular wall structure.^8^ These histopathological findings favour a rare embryological remnant, the PFAA, resulting from incomplete regression of the fifth pharyngeal arch artery during pregnancy. Recognition of this congenital vascular variant is clinically important, as it may mimic acquired aortic pathology and influence both diagnostic interpretation and surgical planning.

PFAA is underrecognized in adults and are commonly misdiagnosed as ductus arteriosus, aortic dissection, or other arch anomalies, complicating the accurate diagnosis of AAA in adults. Giant aneurysms >10 cm in the ascending aorta are very rare, often an asymptomatic life-threatening condition.^9^ Shah *et al*. reported an asymptomatic giant dissecting AAA in a 33-year-old Brazilian man with no significant medical history, discovered incidentally during routine TTE after a diastolic murmur was detected.^10^ Mitsali *et al*. also reported an AAA >10 cm, discovered accidentally in a 72-year-old man who presented with sharp chest pain radiating to the back and shortness of breath.^11^ Although many AAAs remain asymptomatic until complications occur, giant aneurysms may cause compressive symptoms. In this patient, respiratory findings and imaging abnormalities initially led to a suspicion of pulmonary disease, while PFAA mimicked chronic Stanford type A aortic dissection with a false lumen, which underscores the early diagnostic challenges and how congenital vascular anomalies can mimic AAA. In addition, the presence of severe aneurysmal dilation with associated aortic regurgitation necessitated urgent surgical intervention. Valve-sparing root replacement was not considered appropriate due to extensive root involvement and structural valve abnormality. Therefore, complete excision of the diseased aortic root and ascending aorta with composite valved conduit replacement (Bentall procedure) provided definitive treatment and eliminated the risk of rupture while restoring normal aortic valve function.

The coexistence of a chronic congenital anomaly at the top of a massive aneurysm increases the complexity. Chronic PFAA with fibrotic thickening, partial false lumen thrombosis, and reduced wall elasticity can mask the acute clinical signs of dissection, making the diagnosis more difficult.^12,13^ This case emphasizes the importance of maintaining a high index of suspicion for congenital vascular anomalies in patients with atypical imaging findings, particularly when dual-lumen morphology is present. Early surgical intervention remains essential in preventing catastrophic complications and ensuring favourable clinical outcomes.

## Conclusion

This case highlights the diagnostic and surgical challenges of managing a giant ascending aortic aneurysm with dual-lumen morphology. Although radiologic findings initially suggested Stanford type A and DeBakey type I dissection, histopathological examination confirmed aneurysmal degeneration of a persistent fifth aortic arch, a rare congenital vascular anomaly. Despite this discrepancy, the indication for surgery was definitive and based on the extreme aneurysm size and associated severe aortic regurgitation, which independently warranted prompt intervention. The diagnosis of persistent fifth aortic arch did not alter the surgical strategy; although, the potential association of PFAA with certain genetic syndromes should be acknowledged. Depending on future clinical developments, genetic counselling and further testing may be considered if indicated. Definitive management with the Bentall procedure resulted in complete haemodynamic correction and a favourable outcome. This case contributes valuable insight into the natural history of persistent fifth aortic arch and emphasizes that when radiologic findings suggest dissection but clinical or intraoperative features are atypical, histopathological evaluation is essential to establish the definitive diagnosis.

## Supplementary Material

ytag330_Supplementary_Data

## Data Availability

The unidentified patient’s imaging reports and discharge summary were added as supplementary materials.

## Lead author biography

Dr. Abdelrahman is a qualified Physician holding an MD and an MSc in Cardiovascular Health and Rehabilitation. His clinical interests include aortic surgery, structural heart disease, and the perioperative and postoperative management of complex cardiothoracic patients. Dr. Manaa is actively involved in research related to congenital and acquired aortic pathology and is committed to integrating evidence-based practice into surgical care. Outside of medicine, he enjoys reading, travelling, and community health education.

## Notes

**Consent:** The authors confirm that written consent for submission and publication of this case report, including images and associated text has been obtained from the patient, in line with COPE guidance.
