# Supplementary figures and images for: Case report: persistent fifth aortic arch presenting as a giant ascending aortic aneurysm: diagnostic challenge and surgical resolution with Bentall procedure
Source: Eur Heart J Case Rep. 2026 May 12;10(5):ytag330. doi: 10.1093/ehjcr/ytag330 (PMC13192471; doi:10.1093/ehjcr/ytag330)

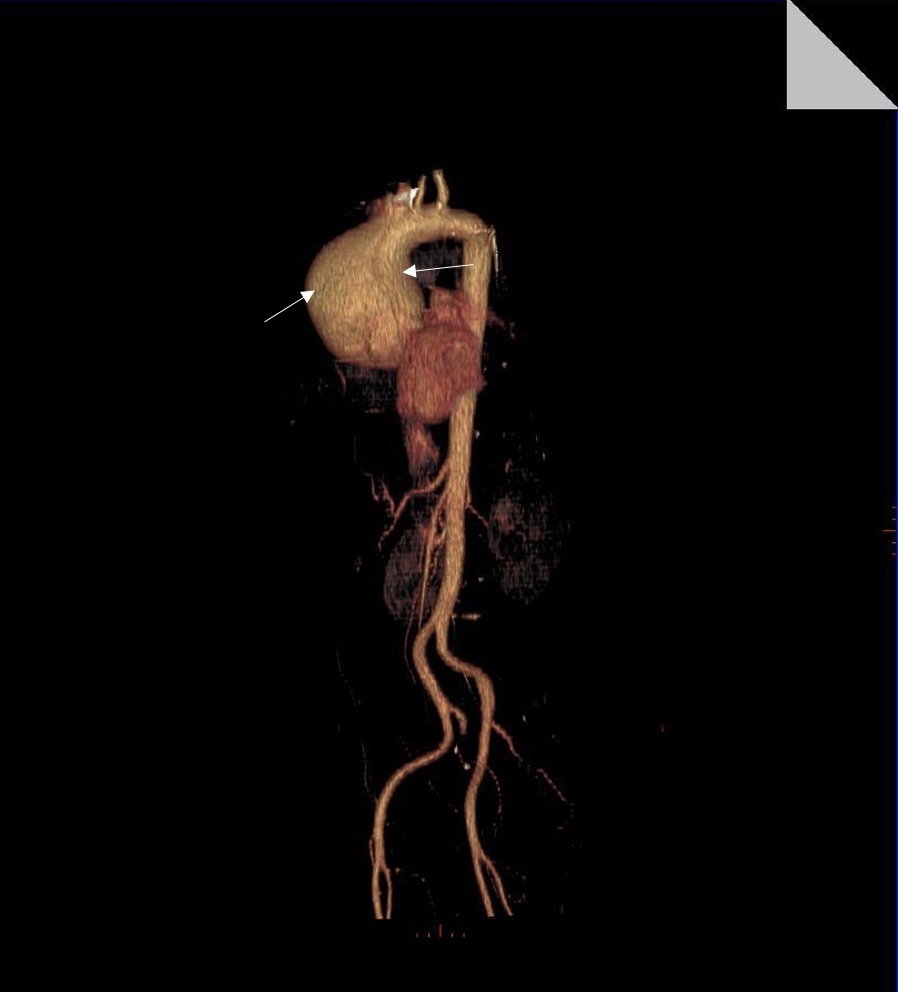

Supplement: ytag330_Supplementary_Data [file ytag330_supplementary_data.zip › Supplementary 3D-1.jpg]

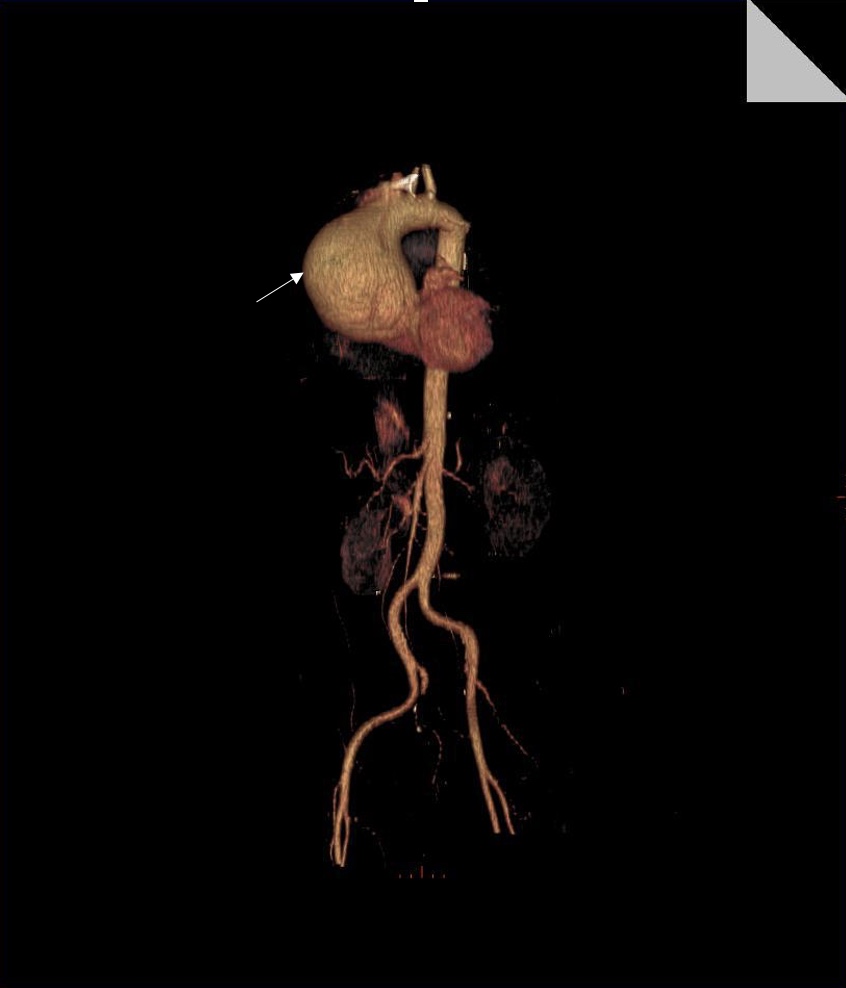

Supplement: ytag330_Supplementary_Data [file ytag330_supplementary_data.zip › Supplementary 3D-2.jpg]

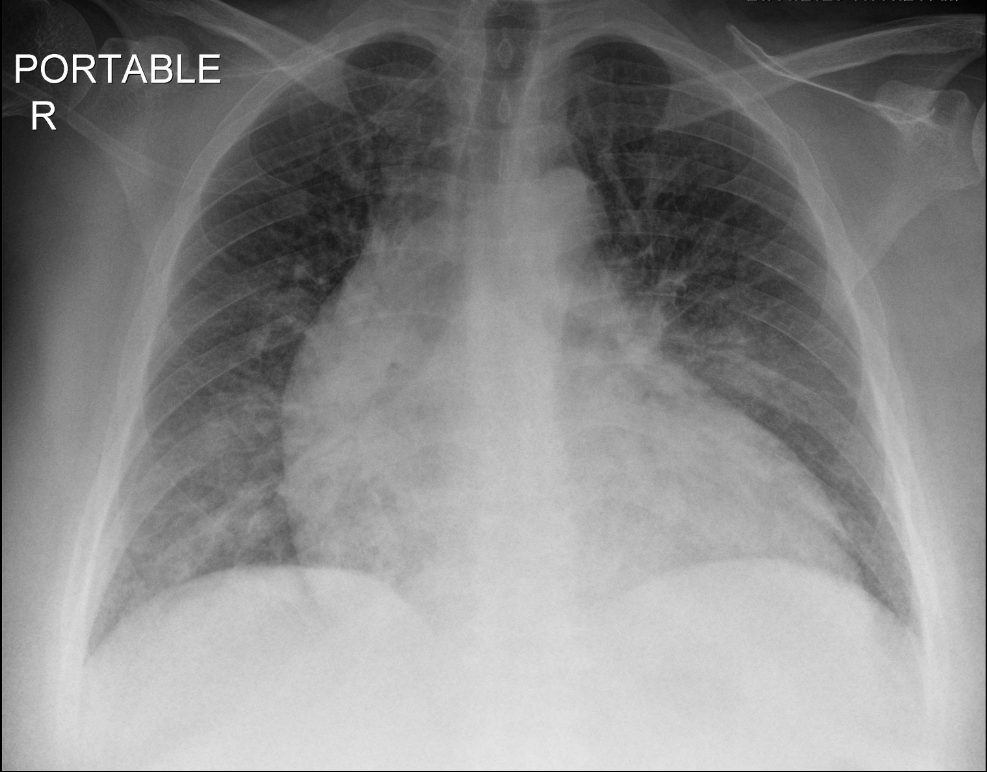

Supplement: ytag330_Supplementary_Data [file ytag330_supplementary_data.zip › Supplementary Chest Xray.png]

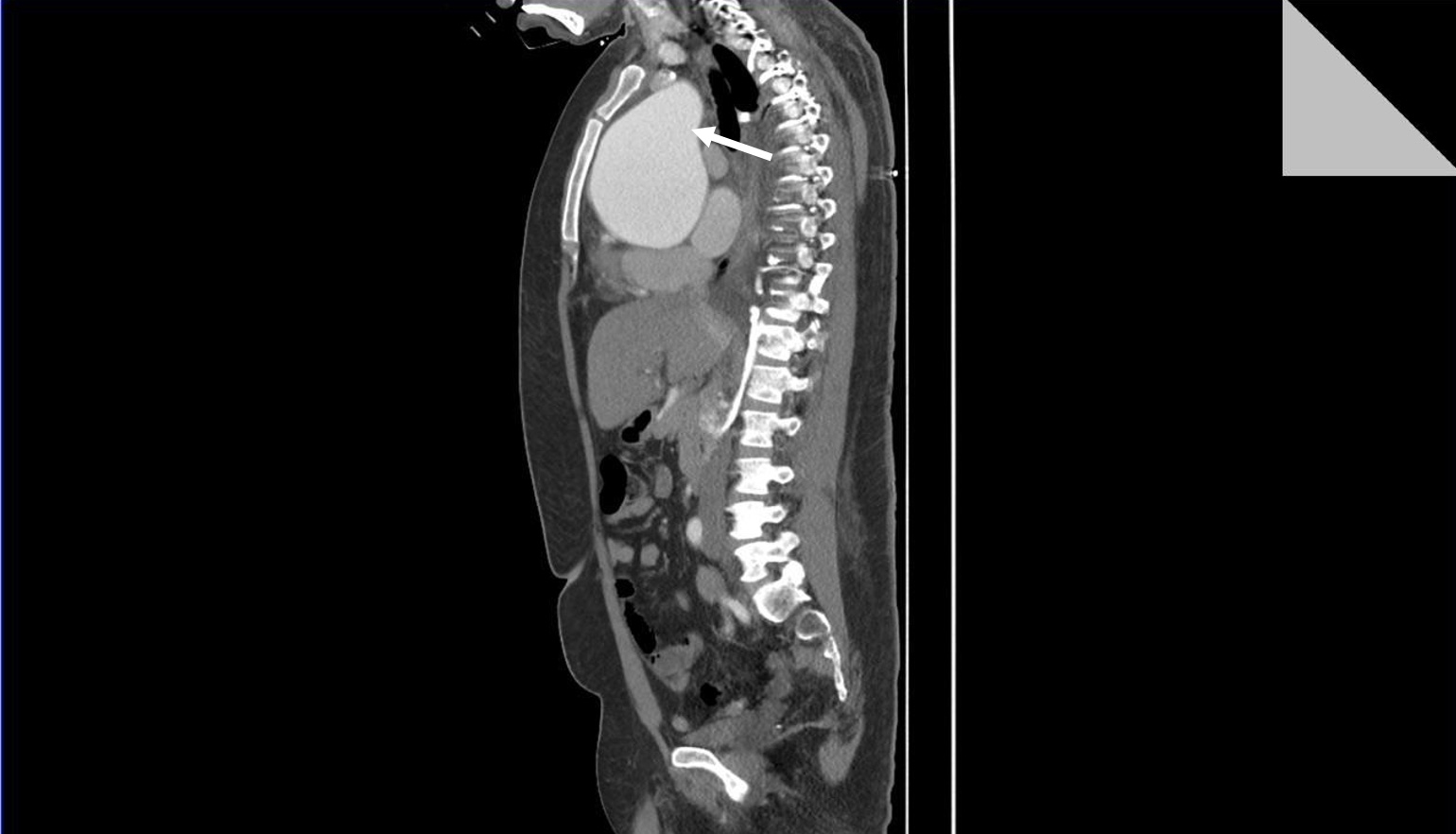

Supplement: ytag330_Supplementary_Data [file ytag330_supplementary_data.zip › Supplementary.jpg]
